# Prevalence, symptom burden, and natural history of deep vein thrombosis in people with advanced cancer in specialist palliative care units (HIDDen): a prospective longitudinal observational study

**DOI:** 10.1016/S2352-3026(18)30215-1

**Published:** 2019-01-29

**Authors:** Clare White, Simon I R Noble, Max Watson, Flavia Swan, Victoria L Allgar, Eoin Napier, Annmarie Nelson, Jayne McAuley, Jennifer Doherty, Bernadette Lee, Miriam J Johnson

**Affiliations:** a Northern Ireland Hospice, Belfast, UK; b Belfast Health and Social Care Trust, Belfast, UK; c Marie Curie Palliative Care Research Centre, Cardiff University, Cardiff, UK; d University of Ulster, Jordanstown, Belfast, UK; e Wolfson Palliative Care Research Centre, Hull York Medical School, University of Hull, Hull, UK; f Hull York Medical School and Department of Health Sciences, University of York, York, UK; g Macmillan Unit, Antrim, UK; h Marie Curie Hospice Belfast, Belfast, UK; i Princess Alice Hospice, Esher, Surrey, UK

## Abstract

**Background:**

The prevalence of deep venous thrombosis in patients with advanced cancer is unconfirmed and it is unknown whether current international thromboprophylaxis guidance is applicable to this population. We aimed to determine prevalence and predictors of femoral deep vein thrombosis in patients admitted to specialist palliative care units (SPCUs).

**Methods:**

We did this prospective longitudinal observational study in five SPCUs in England, Wales, and Northern Ireland (four hospices and one palliative care unit). Consecutive adults with cancer underwent bilateral femoral vein ultrasonography on admission and weekly until death or discharge for a maximum of 3 weeks. Data were collected on performance status, attributable symptoms, and variables known to be associated with venous thromboembolism. Patients with a short estimated prognosis (<5 days) were ineligible. The primary endpoint of the study was the prevalence of femoral deep vein thrombosis within 48 h of SPCU admission, analysed by intention to treat. This study is registered with the ISRCTN registry, number ISRCTN97567719.

**Findings:**

Between June 20, 2016, and Oct 16, 2017, 343 participants were enrolled (mean age 68·2 years [SD 12·8; range 25–102]; 179 [52%] male; mean Australian-modified Karnofsky performance status 49 [SD 16·6; range 20–90]). Of 273 patients with evaluable scans, 92 (34%, 95% CI 28–40) had femoral deep vein thrombosis. Four participants with a scan showing no deep vein thrombosis on admission developed a deep vein thrombosis on repeat scanning over 21 days. Previous venous thromboembolism (p=0·014), being bedbound in the past 12 weeks for any reason (p=0·003), and lower limb oedema (p=0·009) independently predicted deep vein thrombosis. Serum albumin concentration (p=0·43), thromboprophylaxis (p=0·17), and survival (p=0·45) were unrelated to deep vein thrombosis.

**Interpretation:**

About a third of patients with advanced cancer admitted to SPCUs had a femoral deep vein thrombosis. Deep vein thrombosis was not associated with thromboprophylaxis, survival, or symptoms other than leg oedema. These findings are consistent with venous thromboembolism being a manifestation of advanced disease rather than a cause of premature death. Thromboprophylaxis for SPCU inpatients with poor performance status seems to be of little benefit.

**Funding:**

National Institute for Health Research (Research for Patient Benefit programme).

## Introduction

Venous thromboembolism (deep venous thrombosis and pulmonary embolism) is the most common preventable cause of hospital death.^1^ Prevention of hospital-acquired thrombosis is a major health service focus and ranks as the number one hospital strategy for patient safety improvement worldwide.^2^ People with cancer are at particular risk of venous thromboembolism and clinical guidelines recommend pharmacological thromboprophylaxis for all patients with cancer if hospitalised with acute illness.^3^, ^4^ The presence of cancer is an independent risk factor for venous thromboembolism that varies according to primary tumour, stage, and associated cancer-modifying treatments. However, guideline recommendations are extrapolated from thromboprophylaxis trials not done specifically in cancer cohorts and do not consider varying thrombogenicity across the cancer population, particularly as the cancer progresses.^5^ Patients with advanced cancer and a life expectancy of less than 3 months were also excluded systematically from these studies.

Most people with advanced, incurable cancer will be admitted to hospital where they will receive thrombo-prophylaxis routinely.^6^ Only a small proportion will be admitted to a specialist palliative care unit (SPCU), where thromboprophylaxis is a matter of debate, the primary focus of palliative care being symptom control, not survival.^7^ There is a belief that venous thromboembolism is uncommon in the palliative care setting, that data supporting primary thromboprophylaxis are extrapolated from unrepresentative populations (ie, those with prognosis >3 months), and that outcomes from thromboprophylaxis studies (such as radiologically apparent venous thromboembolism), without consideration of symptom impact, are less relevant to people with advanced cancer.^8^, ^9^ Venous thromboembolism in the SPCU setting is considered of clinical relevance only if it confers a patient-reported symptom burden or contributes to distressing symptoms at the end of life. Around the world, few SPCUs (whether in hospital or hospice settings) practice routine thromboprophylaxis.^10^, ^11^, ^12^, ^13^ In the UK, most hospice SPCUs are independent from the National Health Service and therefore lie outside national patient safety initiatives.^14^

**Research in context**

**Evidence before this study**

We searched PubMed for international and national clinical guidelines for the prevention of venous thromboembolism in patients with cancer published between Jan 1, 1980, and Dec 31, 2017, with no language restrictions. Excluding updates, of nine published clinical guidelines (one international and eight national), only one specifically addressed patients receiving palliative care with guidance based on level 5 evidence (grade D recommendation). The UK guideline (National Institute for Health and Clinical Excellence, clinical guideline CG92) is recommended consideration of thromboprophylaxis for potentially reversible causes of increased risk of venous thromboembolism unless the patient was in palliative care. We then searched PubMed for studies published in English between the same dates, with the terms “venous thromboembolism” AND “thromboprophylaxis” or “prophylactic” AND “palliative” OR “advanced cancer” AND “hospice” OR “inpatient”. We identified one randomised controlled trial comparing thromboprophylaxis with the low-molecular-weight heparin (LMWH) nadroparin versus no thromboprophylaxis in patients with advanced cancer in a specialist palliative care unit, but the study recruited only 20 patients. Three clinician surveys identified an inconsistent approach to thromboprophylaxis in specialist palliative care units (SPCUs) and showed that in the acute setting, people with advanced disease are managed differently according to clinical specialty. Before our study, the true prevalence of clinically relevant deep vein thrombosis and its natural history in patients with advanced cancer were unknown.

**Added value of this study**

Our findings showed that approximately one in three people with advanced incurable disease admitted to an SPCU had a femoral deep vein thrombosis, but the he incidence of new thrombosis during the 3 week follow-up was low. Previous venous thromboembolism and being bedbound in the previous 3 months independently predicted deep vein thrombosis. We found no statistically significant association between deep vein thrombosis on admission and survival. Leg oedema was the only venous thromboembolism-relevant sign or symptom associated with deep vein thrombosis. Serum albumin concentration or use of thromboprophylaxis were not related to presence of deep vein thrombosis.

**Implications of all the available evidence**

The high prevalence but low 2-week incidence of femoral deep vein thrombosis in people with advanced cancer on SPCU admission suggests thromboprophylaxis at this stage might be too late. The absence of observed association with survival, or symptoms or signs other than leg oedema questions whether thromboprophylaxis offers clinically meaningful benefit. Our data challenge current international thromboprophylaxis guidelines on a number of counts. First, the findings suggest that the hospital model of care in which patients are risk assessed and given prophylaxis upon admission might be inappropriate for those with advanced cancer and a poor performance status before admission. Second, they raise questions about the optimal timing for introduction of thromboprophylaxis; should it be earlier in advanced disease? Recent work has shown no survival benefit with LMWH prophylaxis in newly diagnosed lung cancer or high-dose LMWH prophylaxis in pancreatic cancer, although high-dose LMWH prophylaxis prevented fatal pulmonary emboli. Third, is thromboprophylaxis of any clinical benefit at all in advanced disease? Is venous thromboembolism merely another manifestation of the inflammatory state of advanced disease, known to be associated with worse survival, and which does the greater damage at this stage of disease?

The provision of thromboprophylaxis for patients receiving palliative care might therefore be determined by place of admission rather than clinical risk. It is unknown whether current practice represents over-treatment (hospital) or under-treatment (SPCU), with hospital patients exposed to the risks of anticoagulation when none is needed, or SPCU patients exposed to the risks of symptomatic venous thromboembolism. We therefore aimed to evaluate the true prevalence of proximal deep vein thrombosis, diagnosed by systematic venous compression ultrasound, in people with advanced, incurable cancer admitted to an SPCU. Secondary outcomes included incidence during admission, associated factors (thromboprophylaxis), and clinical outcomes (symptoms or signs of venous thromboembolism and survival).

## Methods

### Study design and participants

We did a prospective, multicentre, longitudinal, observational prevalence study. Participants were enrolled between June 20, 2016, and Oct 16, 2017. Institutional and ethical (Yorkshire and the Humber–Leeds West Research Ethics Committee) approvals, including for method of consent and management of ultrasound scan results, were granted before recruitment. This study is reported in accordance with the Strengthening the Reporting of Observational Studies in Epidemiology (STROBE) statement. The protocol allowed inclusion of people with advanced non-malignant disease as an exploratory substudy. We report the cancer objectives here only; data relating to people with non-malignant disease are available elsewhere.

Eligible patients were consecutive adults with cancer, aged 18 years or older, admitted to one of five SPCUs ((four hospices and one palliative care unit) in England (n=1), Wales (n=1), and Northern Ireland (n=3; appendix), who were able to give fully informed written consent or, in the absence of mental capacity to provide consent, an appropriate consultee to provide written agreement, and had no physical impediment to femoral vein ultrasound examination (eg, fixed flexion of the hip). There was no upper age limit to participation. If consultee agreement was used, retrospective consent for use of collected data was sought if the participant regained capacity. Patients with a clinician-estimated prognosis of 5 days or less, insufficient mental capacity and no appropriate consultee, or insufficient English or Welsh to provide consent or to comply with study assessments were excluded.

Eligible patients were invited to participate by the admitting clinician. Participants had baseline assessments performed by a research nurse within 48 h of admission including participant demographics, clinical characteristics, venous thromboembolism history, Wells' score,^15^ and blood tests available from routine care. Study assessments were done at baseline and then weekly until discharge or death for a maximum of 3 weeks.

### Procedures

Study outcome measures at baseline were bedside femoral and popliteal vein assessment by ultrasound; Australian-modified Karnofsky performance status (AKPS) score;^16^ clinical examination for signs or symptoms of venous thromboembolism (and new or worsening signs or symptoms at follow-up); known (previously confirmed) venous thromboembolism; bleeding; and medication record including anticoagulation. Noted signs and symptoms of venous thromboembolism were leg oedema and prominent veins; tenderness along the distribution of the deep venous system; calf swelling (circumference at least 3 cm greater than the other calf, measured 10 cm below tibial tuberosity); and pleuritic chest pain or breathlessness. Bleeding was categorised as major or clinically relevant non-major.^17^ Participants were considered at high risk of bleeding if they had thrombocytopenia (platelets <50 × 10^9^ per L), international normalised ratio greater than 1·3, known active gastric or duodenal ulcer, known cerebral metastases, severe and uncontrolled hypertension, renal impairment (creatinine clearance <20 mL/min), or severe liver impairment.

All measures were repeated weekly apart from the medication record. New or worsening clinical symptoms and signs were reported to the clinical team. Overall survival was measured using routinely collected clinical record data from date of study enrolment until date of death from any cause. Deaths recorded using routinely collected clinical record data until 6 weeks after the end of recruitment.

Bilateral femoral and popliteal vein ultrasound scans were undertaken at the bedside by one of five trained research nurses, who was independent to the participant's clinical care. Training occurred at a 2-day ultrasonography course approvied by the Royal College of Physicians, comprising basic ultrasonography physics, practical teaching, and hands-on experience. Staff underwent practical and theory assessments and were required to complete a portfolio of scans before sign-off. Specific to the study, trial nurses had a further day's focused training to optimise skills. The scan process involved identification of the common femoral vein and a compressibility assessment performed at 2 cm increments to the level of the popliteal fossa.

### Outcomes

The primary endpoint of the study was the prevalence of deep vein thrombosis within 48 h of SPCU admission. Secondary endpoints were associated symptoms attributable to deep vein thrombosis, 3-week incidence of new deep thrombosis during admission (with associated symptoms), clinical characteristics associated with the presence of deep vein thrombosis, association between use of anticoagulation and presence of deep vein thrombosis on, and during, admission to an SPCU, impact of deep vein thrombosis on length of stay, and overall survival. With respect to the primary endpoint, one of three possible outcomes was recorded for each scan: no deep vein thrombosis (vein compressible throughout), deep vein thrombosis (vein not compressible at any point), or unevaluable. Further training was provided 3 months into the study to optimise compression technique, improve image quality, and increase the number of evaluable images. We categorised scans done between June 20, and Sept 30, 2016, as early study scans and those done between Oct 1, 2016, and Oct 16, 2017, following the further training of study nurses in bedside ultrasound, as later study scans. All scans were digitally recorded and reviewed by the study radiologist (EN), who was the final arbiter of the presence of deep vein thrombosis or no deep vein thrombosis.

Because screening for deep vein thrombosis is not routinely undertaken in the SPCU, participants and clinicians were masked to ultrasound findings. However, the scan result could be given on request to the treating clinician if there was a clinical suspicion of deep vein thrombosis and the scan had been undertaken within the previous 24 h.

We report the cancer objectives here only; data relating to people with non-malignant disease are available elsewhere.

### Statistical analysis

The sample size calculation was based on a previous study showing bilateral obstruction of venous return in the legs in 17% of SPCU inpatients.^18^ Assuming bilateral obstruction represented more extensive thrombosis, we calculated that a sample size of 217 patients with cancer was needed to estimate the prevalence of proximal lower limb deep vein thrombosis (5% precision; 95% confidence level). Recruitment beyond the sample size for the primary outcome was permitted to improve precision for secondary outcomes.

Participant characteristics are summarised using descriptive analyses with mean (SD; range) or number (%), as appropriate. Prevalence (within 48 h of SPCU admission) is expressed as a percentage with associated 95% CI. Participants with evaluable data for the baseline scan were included in our primary analysis, and the choice of the analysis population was not prespecified.

Univariable logistic regression models were performed to create odds ratios (ORs) and 95% CIs for the following risk factors: age, sex, baseline venous thromboembolism risk factors, use of anticoagulants, AKPS score, venous thromboembolism history, bleeding history, and bleeding risk. All these variables were entered in a multivariable logistic regression model using backward selection with a retention criterion of p value of less than 0·05. Adjusted ORs with 95% CIs were calculated. Missing data were not imputed. Participants with evaluable data for the baseline scan were included in these analyses.

A Kaplan Meier curve was used to compare survival and prevalence of proximal lower limb deep vein thrombosis within 48 h after the patient's admission to SPCU. A log-rank test was used to test for statistical significance. Participants who had not died by the end of survival data collection were right censored.

We did post-hoc sensitivity analyses excluding early scans (those done between June and September, 2016), to account for a technical learning curve, and excluding patients with previous history of deep vein thrombosis. Analyses were done with SPSS version 25.0. This study is registered with the ISRCTN registry, number ISRCTN97567719.

### Role of the funding source

The funder of the study had no role in study design, data collection, data analysis, data interpretation, or writing of the report. MJJ, VLA, and FS had access to all the raw data in the study. EN had access to all scan images. The corresponding author had full access to all the data in the study and had final responsibility for the decision to submit for publication.

## Results

**Figure 1:**
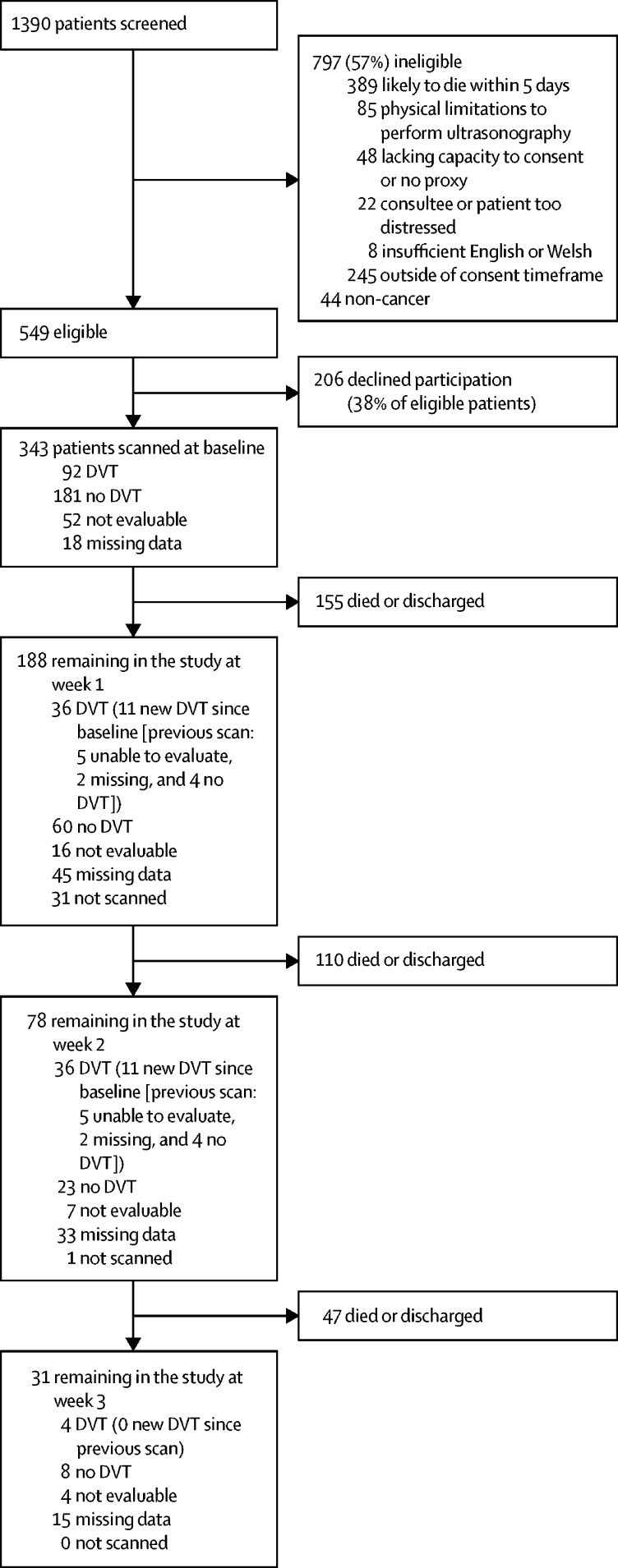
Flow diagram DVT=deep vein thrombosis.

Between June 20, 2016, and Oct 16, 2017, 1390 patients were screened, of whom 343 were recruited (figure 1). 11 participants were admitted more than once during the study period; only the first admission in the study period (n=343) was used for analysis. There was no loss to follow-up.

The most common primary tumour site was lung (20%), followed by upper gastrointestinal, hepatobiliary, or pancreatic (18%), and colorectal (16%) cancer. Most participants (84%) had metastatic disease and 80% had at least one comorbidity (table 1). Almost a quarter (77 [22%] patients) had a history of venous thromboembolism (deep vein thrombosis, 36 [10%]; pulmonary embolism, 55 [16%]). A quarter of participants (82 [24%]) were receiving low-molecular-weight heparin (LMWH) thromboprophylaxis (11 [4%] were on a direct oral anticoagulant; nine [3%] were taking warfarin) and ten (3%) had antithromboembolism stockings alone. 40 (12%) participants were receiving full treatment doses of anticoagulation. Relevant symptoms at baseline included breathlessness (177 [52%] patients), leg oedema (left leg, 147 [43%]; right leg, 137 [40%]), leg pain (left leg, 65 [19%]; right leg, 66 [19%]), haemoptysis (23 [7%]), and chest pain (65 [19%]). Wells' deep vein thrombosis score was likely (≥2) in 174 (51%) participants.

**Table 1:** Baseline characteristics

		**Total population (n=343)**
Age (years)		68·2 (12·8; 25–102)
Sex		
	Male	179 (52%)
	Female	164 (48%)
Family history of venous thromboembolism		
	Yes	49 (14%)
	No or unknown	294 (86%)
Primary cancer		
	Lung	70 (20%)
	Upper gastrointestinal, hepatobiliary, or pancreatic	61 (18%)
	Colorectal	55 (16%)
	Prostate	28 (8%)
	Breast	26 (8%)
	Gynaecological	25 (7%)
	Head and neck	22 (6%)
	Urological	19 (6%)
	Unknown primary	16 (5%)
	Haematological	10 (3%)
	Brain	6 (2%)
	Skin	3 (1%)
	Bone	2 (1%)
Metastatic disease		
	None	56 (16%)
	Yes	287 (84%)
Number of sites of metastases		
	1	98 (28%)
	2	92 (27%)
	3	65 (19%)
	4	23 (7%)
	5	7 (2%)
	6	2 (1%)
Comorbidities		
	None	67 (20%)
	Cardiovascular	141 (41%)
	Gastrointestinal or hepatorenal	90 (27%)
	Respiratory	69 (20%)
	Neurological	48 (14%)
	Diabetes	45 (13%)
	Musculoskeletal	45 (13%)
AKPS score		49 (16·6, 20–90)

Data are mean (SD; range) or n (%). AKPS=Australian-modified Karnofsky performance status.

Ultrasound scans were done in 343 patients on admission. According to the radiologist's assessment, 92 (27%) scans showed deep vein thrombosis and 181 (53%) showed no deep vein thrombosis. 52 (15%) scans were not evaluable, and 18 (5%) were missing. Of 273 patients with evaluable scans and available data, 92 (34%, 95% CI 28–40) had femoral vein thrombosis.

Of the 273 patients with evaluable scans, 28 had a previous history of deep vein thrombosis (data missing for one patient).

Of the 343 patients with cancer, 290 had at least one follow-up scan with a definitive evaluation; 41 of these scans were unevaluable and 12 were done, but the data were missing. Four participants with a scan showing no deep vein thrombosis on admission had a deep vein thrombosis identified subsequently during their admission (maximum 3-weeks' follow-up, figure 1). A further eight participants with unknown deep vein thrombosis status at baseline (because of missing or unevaluable scans) had a deep vein thrombosis.

**Figure 2:**
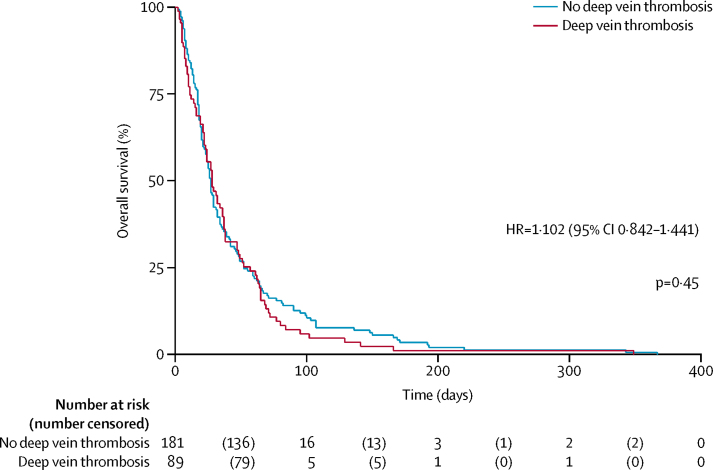
Overall survival in patients with and without deep vein thrombosis on admission Shaded areas represent 95% CIs. HR=hazard ratio.

Table 2 shows the univariable and multivariable regression analyses. Previous venous thromboembolism, being bedbound in the past 12 weeks, and lower limb oedema independently predicted the presence of deep vein thrombosis in the final multivariable model. We noted no association between the use of thromboprophylaxis and the presence of deep vein thrombosis on admission. We found no association between serum albumin concentration and the presence of deep vein thrombosis (mean serum albumin concentration 31·4 mmol/L [SD 6·6] in patients with deep vein thrombosis *vs* 30·6 mmol/L [5·7] in patients without deep vein thrombosis; OR 0·98, 95% CI 0·93–1·03; p=0·43). Mean average survival was 30·55 days (SD 5·65) for patients with deep vein thrombosis versus 31·38 days (6·56) for those without deep vein thrombosis (p=0·432). The presence of deep vein thrombosis on admission was not related to survival (hazard ratio [HR] 1·102 (95% CI 0·842–1·441; p=0·45; figure 2).

**Table 2:** Univariable and multivariable logistic regression analysis

		**No deep vein thrombosis (n=181)**	**Deep vein thrombosis (n=92)**	**Univariable analysis**		**Multivariable analysis**	
				Odds ratio (95% CI)	p value	Odds ratio (95% CI)	p value
**Demographic**							
Age (years)		68·4 (12·8; 25–102)	68·1 (13·5; 29–95)	0·99 (0·98–1·02)	0·86	..	..
Sex							
	Male	95/181 (52%)	50/92 (54%)	1·08 (0·65–1·78)	0·77	..	..
	Female	86/181 (48%)	42/92 (46%)	1 (ref)	..	..	..
Smoking history							
	Current smoker	25/181 (14%)	15/92 (16%)	1·24 (0·58–2·64)	0·85	..	..
	Ex-smoker	86/181 (48%)	43/92 (47%)	1·03 (0·59–1·78)	..	..	..
	Never smoked	70/181 (39%)	34/92 (37%)	1 (ref)	..	..	..
AKPS score		50·0 (15·8; 20–90)	45·2 (16·7; 20–90)	0·98 (0·97–0·99)	0·022	..	..
**Medical history**							
Family history of venous thromboembolism							
	Yes	22/178 (12%)	13/89 (15%)	1·21 (0·58–2·54)	0·61	..	..
	No	156/178 (88%)	76/89 (85%)	1 (ref)	..	..	..
Previous pulmonary embolus							
	Yes	30/181 (17%)	15/92 (16%)	0·98 (0·50–1·93)	0·96	..	..
	No	151/181 (83%)	77/92 (84%)	1 (ref)	..	..	..
Previous deep vein thrombosis							
	Yes	12/181 (7%)	16/91 (18%)	3·00 (1·36–6·66)	0·007	3·00 (1·28–7·00)	0·011
	No	169/181 (93%)	75/91 (82%)	1 (ref)	..	1 (ref)	..
Previous arterial thrombosis							
	Yes	6/181 (3%)	3/91 (3%)	0·99 (0·24–4·07)	0·99	..	..
	No	175/181 (97%)	88/91 (97%)	1 (ref)	..	..	..
Previous venous thromboembolism							
	Yes	33/181 (18%)	29/92 (32%)	2·06 (1·16–3·69)	0·014	2·06 (1·16–3·69)	0·014
	No	148/181 (82%)	63/92 (68%)	1 (ref)	..	1 (ref)	..
**Deep vein thrombosis risk factors within past 12 weeks**							
Acute medical illness							
	Yes	54/181 (30%)	40/92 (43%)	1·81 (1·08–3·05)	0·026	..	..
	No	127/181 (70%)	52/92 (57%)	1 (ref)	..	..	..
Surgery							
	Yes	16/181 (9%)	10/92 (11%)	1·26 (0·55–2·89)	0·59	..	..
	No	165/181 (91%)	82/92 (89%)	1 (ref)	..	..	..
Bedbound in past 12 weeks							
	Yes	27/181 (15%)	27/92 (29%)	2·37 (1·29–4·35)	0·005	2·66 (1·38–5·10)	0·003
	No	154/181 (85%)	65/92 (71%)	1 (ref)	..	1 (ref)	..
**Bleeding within past 6 months**							
Major							
	Yes	7/181 (4%)	2/92 (2%)	0·55 (0·11–2·71)	0·46	..	..
	No	174/181 (96%)	90/92 (98%)	1 (ref)	..	..	..
Non-major							
	Yes	15/181 (8%)	4/92 (4%)	0·50 (0·16–1·56)	0·23	..	..
	No	166/181 (92%)	88/92 (96%)	1 (ref)	..	..	..
Any							
	Yes	22/181 (12%)	6/92 (7%)	0·50 (0·20–1·30)	0·15	..	..
	No	159/181 (88%)	86/92 (93%)	1 (ref)	..	..	..
High risk of bleeding							
	Yes	33/181 (18%)	19/92 (21%)	1·17 (0·62–2·19)	0·63	..	..
	No	148/181 (82%)	73/92 (79%)	1 (ref)	..	..	..
**Venous thromboembolism signs and symptoms at baseline**							
Lower limb oedema (either)							
	Yes	64/180 (36%)	49/92 (53%)	2·07 (1·24–3·44)	0·005	2·08 (1·20–3·60)	0·009
	No	116/180 (64%)	43/92 (47%)	1 (ref)	..	1 (ref)	..
Lower limb pain							
	Yes	33/180 (18%)	23/92 (25%)	1·48 (0·81–2·71)	0·20	..	..
	No	147/180 (82%)	69/92 (75%)	1 (ref)	..	..	..
Chest pain							
	Yes	36/180 (20%)	15/92 (16%)	0·75 (0·39–1·47)	0·41	..	..
	No	144/180 (80%)	77/92 (84%)	1 (ref)	..	..	..
Breathlessness							
	Yes	96/180 (53%)	45/92 (49%)	0·87 (0·52–1·43)	0·57	..	..
	No	84/180 (47%)	47/92 (51%)	1 (ref)	..	..	..
Haemoptysis							
	Yes	13/172 (8%)	4/90 (4%)	0·53 (0·17–1·65)	0·27	..	..
	No	159/172 (92%)	86/90 (96%)	1 (ref)	..	..	..
**Thromboprophylaxis**							
Anticoagulation							
	Yes	43/165 (26%)	26/75 (35%)	1·51 (0·84–2·71)	0·17	..	..
	No	122/165 (74%)	49/75 (65%)	1 (ref)	..	..	..
Antithromboembolism stockings							
	Yes	6/169 (4%)	1/80 (1%)	0·34 (0·04–2·91)	0·32	..	..
	No	163/169 (96%)	79/80 (99%)	1 (ref)	..	..	..

Data are mean (SD; range) or n/N (%), unless otherwise stated. AKPS=Australian-modified Karnofsky performance status.

The presence of leg oedema (either leg) was an independent predictor of deep vein thrombosis (table 2). When analysed by left leg and left leg deep vein thrombosis (table 3), and right leg and right leg deep vein thrombosis (table 4), only left leg oedema was associated with the presence of ipsilateral deep vein thrombosis. There was no association between left leg pain and left leg deep vein thrombosis, or for right leg pain and right leg deep vein thrombosis.

**Table 3:** Patient-reported left leg venous thromboembolism signs and symptoms by deep vein thrombosis on admission

	**No left leg deep vein thrombosis (n=255)**	**Left leg deep vein thrombosis (n=66)**	**Odds ratio (95% CI)**	**p value**
**Left leg oedema**				
Yes	102 (40%)	35 (53%)	1·92 (1·07–3·43)	0·027
No	153 (60%)	31 (47%)	1 (ref)	..
**Left leg pain**				
Yes	43 (17%)	16 (24%)	0·98 (0·48–2·01)	0·96
No	212 (83%)	50 (76%)	1 (ref)	..

**Table 4:** Patient-reported right leg venous thromboembolism signs and symptoms by deep vein thrombosis on admission

	**No right deep vein thrombosis (n=260)**	**Right leg deep vein thrombosis (n=61)**	**Odds ratio (95% CI)**	**p value**
**Right leg oedema**				
Yes	95 (37%)	32 (52%)	1·17 (10·67–2·07)	0·57
No	165 (63%)	29 (48%)	1 (ref)	..
**Right leg pain**				
Yes	48 (18%)	13 (21%)	0·72 (0·33–1·53)	0·39
No	212 (82%)	48 (79%)	1 (ref)	..

At week 1, there were 188 remaining participants of whom seven (4%) reported new lower limb oedema, two (1%) reported new breathlessness, and three (2%) reported new chest pain. Data were missing for 15 (7%). At week 2 there were 80 remaining participants of whom one (1%) reported new lower limb pain, and none reported new breathlessness or new chest pain. Data were missing for four (5%). At week 3 (n=31) one had new leg oedema and one had new breathlessness. In those with an evaluable scan at week 1 (n=96) and week 2 (n=35), there was no difference in new venous thromboembolism-attributable symptoms between those with and those without a deep vein thrombosis (at week 1, two [2%] oedema, two [2%] leg pain, zero breathlessness, and one [1%] chest pain in patients with deep vein thrombosis vs one [1%] oedema, three [5%] leg pain, zero breathlessness, and two [2%] chest pain in patients without deep vein thrombosis; at week 2, one [3%] oedema, zero leg pain, zero breathlessness, and zero chest pain in patients with deep vein thrombosis vs two [2%] oedema, zero leg pain, zero breathlessness, and zero chest pain in patients without deep vein thrombosis.

There were too many missing dates of SPCU discharge to give an estimate on length of stay.

Of 68 early study scans (done between June 2016 and September 2016), 28 (41%) indicated a deep vein thrombosis, 13 (19%) no deep vein thrombosis, 21 (31%) could not be evaluated, and six (9%) were missing. Of 275 later study scans (done between October, 2016, and October, 2017), 64 (23%) indicated a deep vein thrombosis, 168 (61%) no deep vein thrombosis, 31 (11%) could not be evaluated, and 12 (4%) were missing. The difference between early and later study scans for detection of deep vein thrombosis was strongly significant (p<0·001), suggesting a learning curve for scanning. We therefore did a post-hoc sensitivity analysis that excluded the early scans. Of 232 later study scans with a definitive evaluation, 64 (28%, 95% CI 22–34) showed femoral deep vein thrombosis.

In a further sensitivity analysis excluding patients with a past history of deep vein thrombosis (n=28), 75 of 244 patients (31%, 95% CI 25–37) had a femoral vein thrombosis. If early scans in this group were excluded, 52 of 209 patients (25%, 19–31) had a femoral vein thrombosis.

## Discussion

In this prospective longitudinal observational study, bedside compression ultrasonography identified femoral deep vein thrombosis in about a third of eligible people with advanced cancer admitted to an SPCU, with post-hoc analyses suggesting apparently new diagnoses of deep vein thrombosis (ie, in patients with no history of deep vein thrombosis, using optimised scanning technique) in a quarter of patients. Although iliofemoral deep vein thrombosis indicates a large clot burden, our findings showed no difference in relevant symptoms between participants with or without deep vein thrombosis apart from an association with lower limb oedema. Participants with deep vein thrombosis were more likely to have a history of venous thromboembolism, or to have been bedbound for any reason during the previous 3 months, than were those without deep vein thrombosis. Notably, there was no association between the presence or absence of deep vein thrombosis and thromboprophylaxis use. No statistical association was seen between the presence of deep vein thrombosis and serum albumin concentration, despite previous studies suggesting such an association.^19^ However, the previous data were recorded in a much healthier population followed up for a mean 723 days. Our findings show no difference in survival between patients with or without deep vein thrombosis. The numbers of participants developing new deep vein thrombosis subsequent to admission was low and conferred no additional symptom burden, although these findings should be treated with caution in view of the small numbers of patients.

More than half of all screened patients were ineligible (61%), mainly because death was expected within 5 days. Of the 549 eligible patients, 206 (38%) declined participation. Ethical approval did not permit the recording of demographics of non-consenting patients, although trial nurses reported that the population was similar to patients who consented. However, enrolled participants had similar demographics to those reported in the National Council for Palliative Care Minimum Data Set for SPC inpatient units with respect to sex, age, primary diagnosis, and metastatic burden.

Apart from limb oedema, our findings showed no effect of femoral vein thrombosis on experience of venous thromboembolism-related symptoms, and no effect of thromboprophylaxis on deep vein thrombosis risk. Thromboprophylaxis might therefore confer no benefit over analgesia or other appropriate control measures for deep vein thrombosis symptoms in patients with cancer admitted to an SPCU. Recruited participants had a mean AKPS score of 49 (a value of 50 represents the need for considerable assistance and frequent medical care),^16^ most had metastatic disease and at least one comorbidity, and mean length of survival was only 44 days. These characteristics not only demonstrate a functionally dependent population with advanced stage of illness but also one previously unrepresented in thromboprophylaxis studies, in which life expectancy of less than 3 months was invariably an exclusion criterion.

This was a pragmatic multicentre study done in SPCUs across the UK, with broad entry criteria. Clinical studies in this environment are notoriously challenging, yet HIDDen completed target recruitment ahead of time, allowing analysis of adequately powered data. Unlike previous venous thromboembolism studies, which have been difficult to contextualise in advanced cancer, we used pre-agreed outcome measures of relevance to patients and treating clinicians.^8^ To optimise recruitment, compression ultrasonography was done at the bedside by trained research nurses and independently validated by a consultant radiologist. Every effort was made to ensure the quality of scans and for this reason additional training was provided to the research nurses at month 3 of the study. Specifically, focus was placed on improved compression technique and increased number of images recorded for radiologist review. Furthermore, we did a sensitivity analysis excluding the first 3 months' scans to allow for a learning curve. However, we acknowledge that these results are likely to under-represent the prevalence of venous thromboembolism because the analysis will have omitted distal deep vein thrombosis and pulmonary embolus. In patients with cancer, distal deep vein thrombosis is associated with an increased risk of residual vein thrombosis, an established risk factor for recurrent deep vein thrombosis.^20^ Furthermore, identification of fresh deep vein thrombosis by compression ultrasonography in the presence of a recent deep vein thrombosis is notoriously challenging and it is for this reason that we omitted patients with history of deep vein thrombosis (proximal or distal) in our additional sensitivity analysis.^21^ Additionally, 5% of scans were missing. These were unlikely to be missing completely at random because participants, although they consented to participate, might then have declined a scan if they felt less well. This missing group might therefore have included at least some patients more likely to have a deep vein thrombosis because of poorer performance status, and thus the prevalence might be underestimated. This issue is greater for development of deep vein thrombosis during follow-up, where missing or unevaluable scans are greater. However, data were more complete for symptom report, and there were no between-group differences in new symptoms that could be attributable to thromboembolism.

The study sample size was calculated to provide adequate power for estimates of prevalence and not for survival, and the data are observational. A randomised controlled trial of thromboprophylaxis with survival as the primary endpoint, using data from an epidemiological model of venous thromboembolism-related hospital-acquired deaths, would need a sample size of 72 000— unlikely to be feasible or to provide value for information in this patient population.^22^

Our data challenge current recommendations for prevention of venous thromboembolism prevention in advanced cancer.^4^ Strategies to prevent hospital-acquired thrombosis remain a global health priority and patients with advanced cancer would seem an ideal patient group to target because they are highly thrombotic and 88% will have an average of five hospital admissions in their last year of life.^23^ A prospective observational study of 22 SPCUs in France identified a 9·8% (95% CI 8·3–11·6) incidence of clinically relevant bleeding in 1199 patients with cancer.^24^ Multivariate analysis suggested an association between clinically relevant bleeding and pharmacological thromboprophylaxis (HR 1·48, 95% CI 1·02–2·15; p=0·04).^24^ It would seem expedient to minimise the risk of harm by avoiding the use of thromboprophylaxis unless there will be a clear net benefit. Our data show a high prevalence of femoral deep vein thrombosis at the point of admission to the SPCU, making the issue of thromboprophylaxis a moot point. Since our data suggest these deep vein thromboses confer a minimal symptom burden with no evidence that they shorten life, rethinking the utility of pharmacological thromboprophylaxis in this population would seem reasonable. However, use of thromboprophylaxis is dependent upon place of admission, and is much less likely in SPCU inpatients than in those admitted to an acute hospital setting.

Whether these data can be extrapolated to the acute setting is debatable. The advanced cancer population in this study were defined by SPCU admission. The factors influencing the clinical decision to admit to the SPCU setting are unknown but will be more complex than extent of disease or prognosis, and are likely to include patient preferences, performance status, and clinician judgment regarding potential reversibility of any deterioration. Qualitative research offers some explanation for discrepant practice between hospitals and SPCUs. First, although around half of people admitted to SPCUs have evidence of obstruction to lower limb venous flow measured by light reflection rheography (17% bilateral)^18^ and 50% have venous thromboembolism at post mortem, palliative care teams perceive that venous thromboembolism is not a common clinical problem.^9^ Clinicians might misattribute symptoms to other pathologies such as lymphoedema, hypoalbuminaemia, or cellulitis (for deep vein thrombosis), and anaemia, pneumonia, pleural effusion, heart failure, lymphangitis, and lung metastases (for pulmonary embolism). Conversely, perhaps despite a high prevalence, venous thromboembolism-related symptoms contribute a small proportion to the overall symptom burden of people with advanced incurable illness. In our study, a significant predictor for deep vein thrombosis was being bedbound for any reason within the past 12 weeks. This characteristic not only delineates a subpopulation of people with advanced disease with a potentially poorer prognosis, but also suggests that their thrombotic insult might have been experienced before admission as part of an inevitable decline rather than the cause of it. This suggestion raises the question whether thromboprophylaxis should be started before developing advanced disease. However, LMWH prophylaxis in patients with newly diagnosed lung cancer^25^ or high-dose LMWH prophylaxis in patients with pancreatic cancer^26^ did not improve survival, although high-dose LMWH prophylaxis prevented fatal pulmonary emboli. People with advanced cancer admitted to an acute hospital setting likely represent a broader population with respect to performance status and prognosis; therefore, without a clearer understanding of the demographics, we cannot apply these findings beyond the SPCU setting. However, our study offers new insights into the utility and appropriateness of thromboprophylaxis strategies for patients with cancer nearing the end of life. The numbers of participants receiving anticoagulation for previously diagnosed deep vein thrombosis or pulmonary embolism were too small to draw any conclusions about any benefit from secondary prevention of venous thromboembolism for symptom control or survival.

These novel data show that approximately a third of patients admitted to SPCUs with advanced cancer and who were not expected to die within 5 days had a femoral deep vein thrombosis. Deep vein thrombosis was not associated with thromboprophylaxis, survival, or symptoms other than leg oedema. Findings are consistent with venous thromboembolism being a manifestation of advanced disease rather than a cause of premature death. Thromboprophylaxis for SPCU inpatients with poor performance status seems to be of little benefit.

## Data sharing

Data can be accessed by contacting the corresponding author.
