# Correction to *Lancet Haematol* 2019; 6: e79–88

**DOI:** 10.1016/S2352-3026(19)30084-5

**Published:** 2019-05-21

*White C, Noble SIR, Watson M, et al. Prevalence, symptom burden, and natural history of deep vein thrombosis in people with advanced cancer in specialist palliative care units (HIDDen): a prospective longitudinal observational study.* Lancet Haematol *2019; **6:** e79–88*—In figure 1, the number of ineligible patients should have been 825 (59%), among which 28 non-cancer; the number of eligible patients should have been 565, of whom 359 were recruited and 16 excluded because they were repeat admissions. The second sentence of the results section should have been “16 participants were admitted more than once during the study period”. These corrections have been made to the online version as of May 21, 2019.

