# Supplementary material for: Prevalence, symptom burden, and natural history of deep vein thrombosis in people with advanced cancer in specialist palliative care units (HIDDen): a prospective longitudinal observational study
Source: Lancet Haematol. 2019 Jan 29;6(2):e79–88. doi: 10.1016/S2352-3026(18)30215-1 (PMC6352715; doi:10.1016/S2352-3026(18)30215-1)
Supplement: Supplementary appendix [file mmc1.pdf]

THE LANCET
Haematology

Supplementary appendix

This appendix formed part of the original submission and has been peer reviewed. We post it as supplied by the authors.

Supplement to: White C, Noble SIR, Watson M, et al. Prevalence, symptom burden, and natural history of deep vein thrombosis in people with advanced cancer in specialist palliative care units (HIDDen): a prospective longitudinal observational study. *Lancet Haematol* 2019; **6:** e79–88.

Recruitment sites and number of patients recruited

Recruitment site	Principal Investigator	Number of patients recruited
Princess Alice, Esher, Kent	Bernadette Lee	70
Macmillan Unit, Antrim	Jayne McAuley	81
Marie Curie Hospice Cardiff	Simon Noble	58
Northern Ireland hospice	Clare White	129
Marie Curie Hospice Belfast	Jennifer Doherty	49
